# Etiquette

**Published:** 1881-08

**Authors:** 


Etiquette.—" Why is it that cordiality, and the same courtesy and freedom of intercourse are not found generally among medical men as in other callings ?

A few men in the profession here seem to assume an air of *hauteur* and superciliousness in their bearing towards their brethren, which is incompatible with ordinary good manners. Some even passing and repassing without recognition of one another.

You have been in the profession somewhat longer than I have, and perhaps you can account for such conduct from your personal observation or experience ?

Yours, truly.

C."

A. We have been a member of the medical profession for more than a third of a century, fifteen years of which have been passed in Baltimore. We have never, either here or elsewhere, received any other than polite and courteous treatment from well-bred gentlemen.

Nor have we ever had personal difficulity nor anything more than mere temporary estrangement from misunderstanding or misapprehension *with any gentleman* in the profession. We can go farther and add that the only enemies we have had are persons whom we have aided, directly or indirectly, in bringing from comparative obscurity into the little prominence they have. But this is in accordance with the old saw, "Serve a man, and make an enemy." There are, however, some honorable exceptions, even among this latter class; and we are always proud to rank them among our best and warmest friends. But *they* were gentlemen, both by birth and education, and *could not* have been other than what they are. Should our worthy correspondent trouble himself to look into the antecedents of those to whom he alludes, he will doubtless see the truth of the maxim, that "it is impossible to make a silk purse out of a sow's ear." A man of gentle birth, instincts and proper culture, will remain a gentleman even though he should become a physician! And no amount of medical degrees or diplomas *can transform* a clown into a gentleman. We always endeavor to treat this latter class, whether found in the profession or elsewhere, with the same philosophy "My uncle Toby" exercised toward the fly, and we would advise our correspondent to a similar course.

